# Offspring sex affects the susceptibility to maternal smoking-induced lung inflammation and the effect of maternal antioxidant supplementation in mice

**DOI:** 10.1186/s12950-020-00253-5

**Published:** 2020-08-05

**Authors:** Baoming Wang, Yik Lung Chan, Shengyu Zhou, Sonia Saad, Hui Chen, Brian G Oliver

**Affiliations:** 1 Faculty of Science, School of Life Sciences, University of Technology Sydney, Sydney, NSW 2007 Australia; 2 Respiratory Cellular and Molecular Biology, Woolcock Institute of Medical Research, The University of Sydney, Sydney, NSW 2037 Australia; 3 School of Nursing, Shandong University, Jinan, 250012 Shandong China; 4 Department of Pulmonary Medicine, Qilu Hospital of Shandong University, Jinan, 250012 Shandong China; 5 Renal Group Kolling Institute, Royal North Shore Hospital, St Leonards, NSW 2065 Australia

**Keywords:** Antioxidant, Sex differences, Inflammasome, Mitophagy

## Abstract

**Background:**

Cigarette smoke exposure (SE) during pregnancy is the largest modifiable risk factor for the development of lung disorders in offspring. We have previously shown that maternal L-Carnitine treatment can reduce the adverse impacts of maternal SE on renal and brain disorders in offspring. Here, we investigated the effect of maternal L-Carnitine supplementation on lung inflammatory pathways, autophagy, and mitophagy markers in the offspring in response to maternal SE.

**Methods:**

Female BALB/c mice (8 weeks) were exposed to cigarette smoke for 6 weeks prior to mating, during gestation and lactation. Some of the SE dams were given L-Carnitine supplementation (1.5 mM in drinking water, SE + LC) during gestation and lactation. Lungs from the offspring were studied at birth and adulthood (13 weeks).

**Results:**

At birth, in male offspring, there were increased levels of inflammatory markers (phosphorylated(p)-ERK1,2, p-P38 MAPK, p- NF-κB), and inflammasome marker (NLRP3), as well as mitophagy fission marker Drp-1 and autophagosome marker (LC3A/B-II) in the lung. Maternal L-Carnitine supplementation significantly reduced NLRP3 level. In contrast, maternal SE only increased IL1-β in female offspring, which was reversed by maternal L-Carnitine supplementation. At 13 weeks, there was an increase in LC3A/B-II and p- NF-κB in the male SE offspring with reduced p-JNK1,2, which were partially normalised by maternal L-Carnitine treatment. Female offspring were not affected by maternal SE at this age.

**Conclusion:**

Maternal SE had adverse impacts on the male offspring’s lung, which were partially alleviated by maternal L-Carnitine supplementation. Females seem to be less affected by the adverse effects of maternal SE.

## Background

Smoking during pregnancy is a major cause of maternal and newborn morbidity and mortality [1], with pulmonary diseases being a major adverse outcome [2, 3]. In-utero smoke exposure (SE) reduces lung function in human newborns [4, 5]. Animal models have shown a decreased number of saccules, septal crests, and decreased elastin fibres in foetuses [6] and suckling pups [7], as well as increased airway thickness, collagen deposition, inflammation, and airway hyper-responsiveness due to intrauterine SE [8–10].

In humans, certain respiratory diseases, including chronic obstructive pulmonary disease (COPD), occur disproportionately in males and females [11]. The common pathophysiological process includes increased inflammation, oxidative stress, impaired mitochondrial renewal (mitophagy), and cellular self-cleaning mechanism (autophagy) [12]. In keeping with this, our previous murine studies found that the changes in inflammation, oxidative stress, mitophagy, and autophagy have a marked sex difference in the offspring’s brain and kidney following in-utero SE, wherein female offspring are less affected from such adverse effects [13, 14].

Previously, it has only been demonstrated that maternal SE causes lung inflammation in male offspring [15]. Other studies show that prenatal SE can differentially affect DNA methylation in cord blood or lungs in males and females in both human and animal studies. For example, the gene encoding insulin-like growth factor (IGF)-1, a growth factor involved in lung development, was found to be methylated in the babies from smokers, and methylation was most pronounced in male children [16, 17]. In an animal study, CpG site-specific hypo- and hypermethylation of *Igf1* exons was observed in the lungs from male and female offspring, respectively. However, protein transcription of IGF-1 is reduced in male offspring, even without in utero SE, which may partially explain sex differences in the susceptibility of developing certain respiratory disorders [18]. The mechanism by which in-utero causes differential DNA methylation between males and females is not known, but a recent study in T cells has demonstrated that the parental source of the X chromosome (male versus female) affects the methylation of the X chromosome in offspring [19], suggesting that the sex chromosomes may be involved.

Given these known differences, we hypothesised that sex differences also exist regarding the effects of in-utero SE on other pulmonary changes.

The regulation of inflammation involves several signalling pathways, such as nuclear factor kappa-light-chain-enhancer of activated B cells (NF-κB) and mitogen-activated protein kinase (MAPK) pathways [20]. Three well-characterised subfamilies of MAPK include the extracellular signal-regulated kinase (ERK)1/2, Jun N-terminal kinase (JNK) stress-activated protein kinase, and p38 [21]. NF-κB is often regarded as the master controller of inflammation [22]. Inflammatory response requires a considerable amount of energy derived from the mitochondria [23], whereas mitochondrial function is often compromised during this process. There is a close relationship between the activation of the nucleotide-binding domain and leucine-rich repeat-containing family pyrin domain containing 3 (NLRP3) inflammasome (increasing IL-1β activity) and mitochondrial dysfunction [24]. Thus, the inflammasome is regarded as the bridge between inflammatory response and subsequent mitochondrial damage [24], including oxidative stress [24] and mitochondrial DNA impairment [25]. This has been observed in conditions like COPD but has not been investigated in the setting of maternal SE [26].

Mitophagy is the autophagic elimination of injured mitochondria, which is regulated by fusion and fission [27]. The balance between fusion and fission is essential to mitochondrial integrity. Fission is to separate damaged mitochondrial fragments from the healthy part, while fusion is to generate a new mitochondrion from two healthy mitochondrial fragments [28, 29]. We have observed dysregulated mitophagy in the brain and kidney caused by maternal SE which was associated with organ pathology [13, 30]; however, whether this also occurs in the lung is unknown.

In-utero SE results in considerable foetal oxidative stress and inhibits the endogenous antioxidant activity [31]. Therefore, improving antioxidant ability may alleviate the adverse effects of maternal SE. L-Carnitine has been shown to attenuate age-related disorders by reducing oxidative stress and increasing antioxidant capacity in rats [32, 33]. A clinical study also showed that L-Carnitine supplementation can suppress serum levels of inflammatory cytokines in humans [34]. We have shown that maternal L-Carnitine supplementation during pregnancy and lactation can alleviate brain [13] and renal dysfunction [35] in offspring from the SE mothers. As such, this approach may also ameliorate the adverse impact of maternal SE on lung health in the offspring.

Given the known differences in the susceptibility of developing lung diseases between males and females [36], we hypothesised that in-utero smoke exposure would result in chronic hyperactivation of inflammatory markers and dysregulated autophagy and mitophagy in male offspring, but not in female offspring. Maternal L-carnitine may ameliorate the adverse impact of maternal SE on the offspring’s lung.

## Results

### Effect of maternal SE on body weight

At postnatal(P)1, both male and female offspring from the SE dams appeared smaller than the SHAM offspring (Table 1). Maternal L-Carnitine supplement during gestation and lactation increased the birth weight of both male and female offspring (*P* < 0.05 vs SE). There were no differences in body weight among the 3 groups at 13 weeks for both males and females (Table 1). There was a non-significant decrease in the litter size from the SE dams, and the male-female ratio was unaffected Table 2).

**Table 1:** Body weight of the offspring at different ages

Male offspring	Day 1			13 weeks		
	SHAM	SE	SE + LC	SHAM	SE	SE + LC
	*n* = 8	*n* = 9	*n* = 8	*n* = 8	*n* = 7	*n* = 8
Body weight (g)	1.53 ± 0.29	1.30 ± 0.12	1.62 ± 0.2*	25.6 ± 0.9	24.7 ± 0.9	25.7 ± 1.24
Female offspring	SHAM	SE	SE + LC	SHAM	SE	SE + LC
	n = 8	n = 6	n = 8	n = 8	n = 8	n = 8
Body weight (g)	1.48 ± 0.38	1.21 ± 0.06	1.68 ± 0.19*	22.0 ± 1.2	20.7 ± 1.0	21.0 ± 0.7

Results are expressed as mean ± SEM. Data were analysed by one-way ANOVA followed by Tukey’s post hoc tests. *P < 0.05, compared with the SE offspring at the same age. SE, maternal smoke exposure; SE + LC, maternal smoke exposure with L-Carnitine supplement

**Table 2:** Litter demographics

	Sham	SE	SE + LC
Litter size (pup / litter)	6.2 ± 0.8	5.3 ± 1.0	6.0 ± 1.0
Male pup / litter	3.5 ± 0.5	2.9 ± 0.7	3.0 ± 0.6
Female pup / litter	2.6 ± 0.6	2.4 ± 0.6	3.0 ± 0.5

Results are expressed as Mean ± SEM. *n* = 9–12. The data were analysed by One-way ANOVA followed by Turkey’s post hoc tests

### Effect of maternal SE on lung p-ERK, p-p38, p-JNK, and p- NF-κB in the offspring

**Fig. 1:**
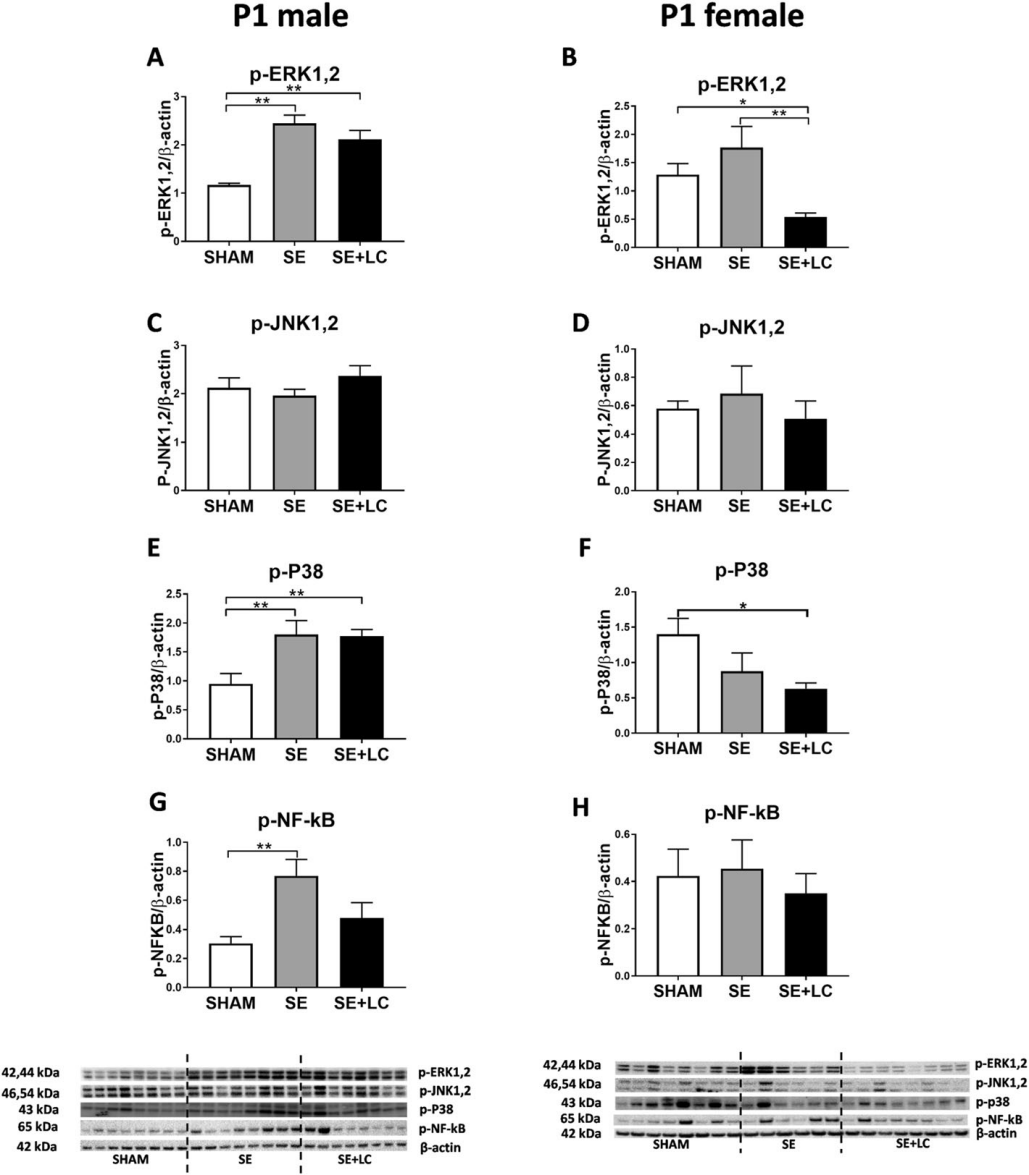
Lung p-ERK1,2, p-JNK1,2, p-p38 and p- NF-κB in the offspring at P1. Protein levels of p-ERK1,2 **a**, **b**, p-JNK1,2 **c**, **d**, p-p38 **e**, **f** and p- NF-κB **g**, **h** in the lung of the male and female offspring at P1. Results are expressed as means ± SE, (male *n =* 8, female, *n =* 6–8). Data were analysed by one-way ANOVA followed by Tukey’s post hoc tests. *P < 0.05, **P < 0.01, *** *P* < 0.001, ****P < 0.0001. ERK, extracellular signal-regulated kinase; JNK, c-JUN N-terminal kinase; p38, p38 Mitogen-activated protein kinase; NF-κB: Nuclear factor-kB. SE, maternal smoke exposure; SE + LC, maternal smoke exposure with L-Carnitine supplement

At P1, maternal SE significantly increased the levels of p-ERK1,2 (*P* < 0.01 vs SHAM, Fig. 1a), p-P38 (*P* < 0.01 vs SHAM, Fig. 1e) and p-NF-κB (*P* < 0.01 vs, SHAM, Fig. 1g) in the male offspring. Only p- NF-κB appeared to be partially reversed by maternal L-Carnitine treatment without statistical significance (Fig. 1g). In the female offspring, maternal SE did not significantly affect p-ERK1,2, p-JNK1,2, p-p38, or NF-κB levels, whereas maternal L-Carnitine supplementation significantly reduced p-ERK1,2 (*P* < 0.05 vs SHAM, *P* < 0.01 vs SE, Fig. 1b) and p-P38 (*P* < 0.05 vs SHAM, Fig. 1f) levels.

**Fig. 2:**
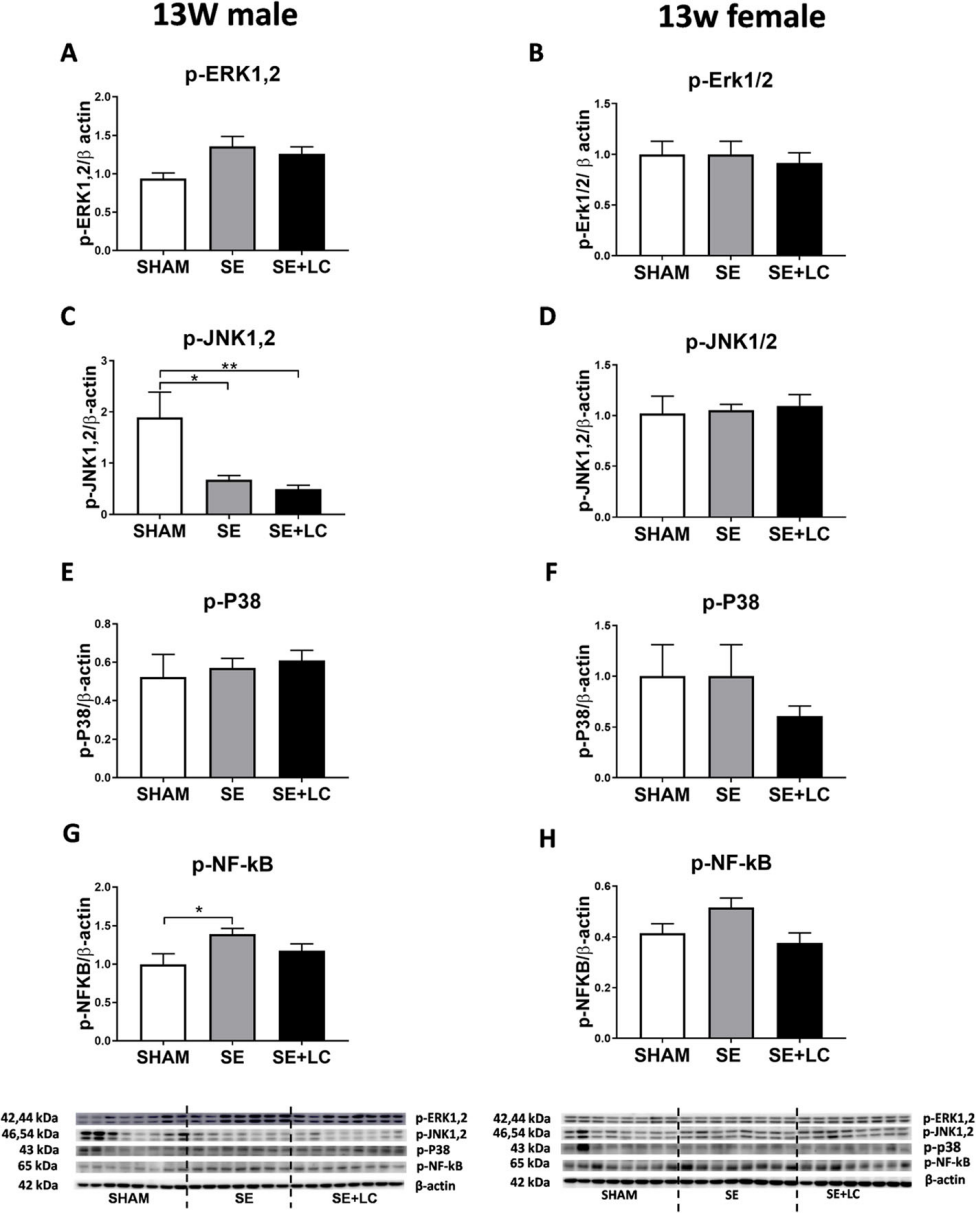
Lung p-ERK1,2, p-JNK1,2, p-p38 and p- NF-κB in the offspring at 13 weeks. Protein levels of p-ERK1,2 **a**, **b**, p-JNK1,2 (C, D), p-p38 **e**, **f** and p- NF-κB **g**, **h** in the lung of the male and female offspring at 13 weeks. Results are expressed as means ± SE, (male *n =* 7*–*8, female *n =* 8). Data were analysed by one-way ANOVA followed by Tukey’s post hoc tests. *P < 0.05, **P < 0.01. ERK, extracellular signal-regulated kinase; JNK, c-JUN N-terminal kinase; p38, p38 Mitogen-activated protein kinase; NF-κB: Nuclear factor-kB. SE, maternal smoke exposure; SE + LC, maternal smoke exposure with L-Carnitine supplement

At 13 weeks, p-JNK1,2 level was lower, and p- NF-κB was higher in the male offspring (*P* < 0.05 vs SHAM offspring, Fig. 2c, g), which was not affected by maternal L-Carnitine supplementation. In the adult females, neither maternal SE nor maternal L-Carnitine supplementation had any effect on the abovementioned proteins (Fig. 2).

### Effect of maternal SE on lung NLRP3 and IL1-β levels in the offspring

**Fig. 3:**
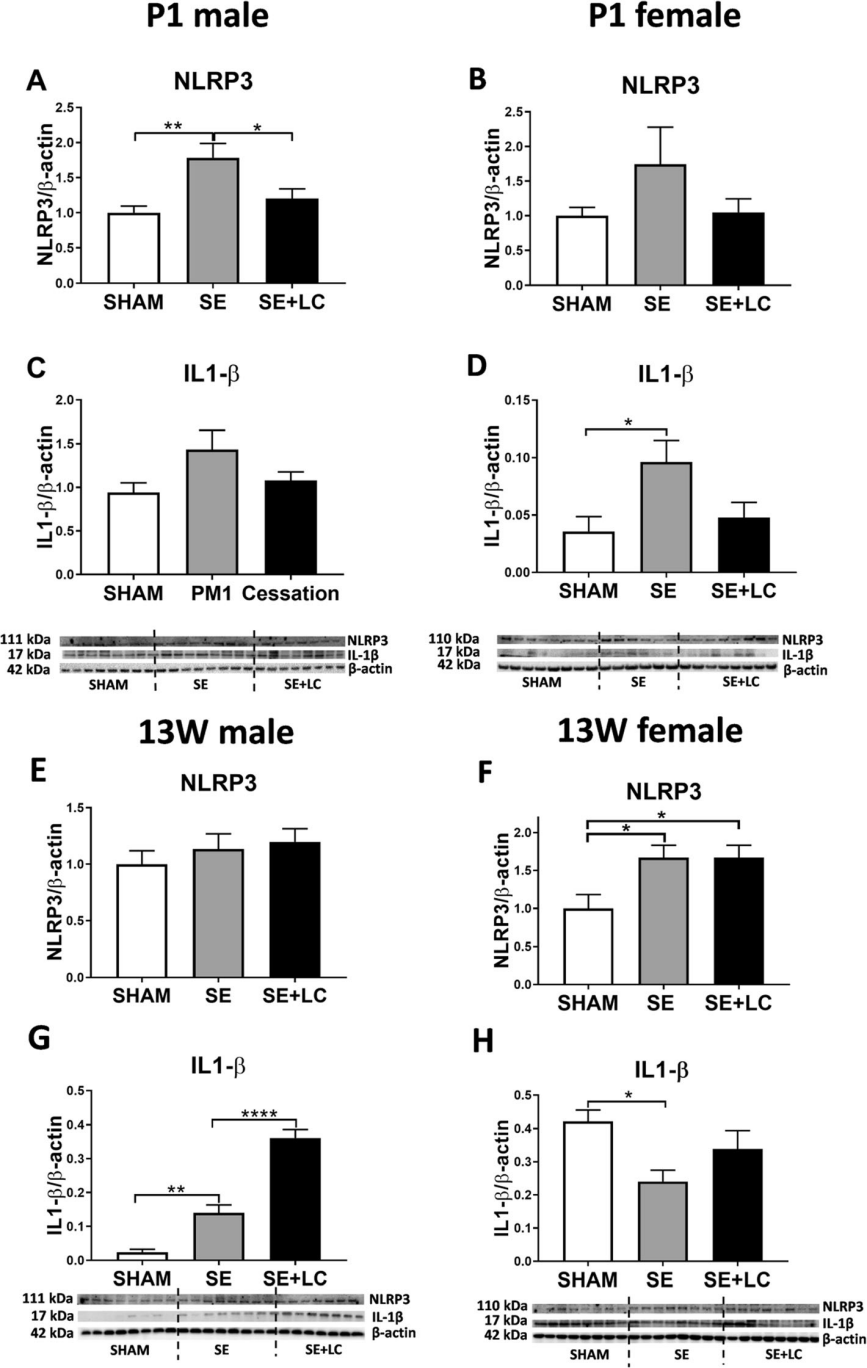
Lung inflammasome markers NLRP3 and IL-1 β in the offspring at P1 and 13 weeks. Protein levels of NLRP3 **a**, **b** and IL-1β **c**, **d** in the lung of male and female offspring at P1. Protein expression of NLRP3 **e**, **g** and IL-1β **f**, **h** in the lung of male and female offspring at 13 weeks. Results are expressed as means ± SE (male *n =* 8, female *n =* 6–8). Data were analysed by one-way ANOVA followed by Tukey’s post hoc tests. *P < 0.05, **P < 0.01, ****P < 0.0001. NLRP3, nucleotide-binding domain and leucine-rich repeat-containing (NLR) family pyrin domain containing 3; SE; maternal smoke exposure; SE + LC, maternal smoke exposure with L-Carnitine supplement

In P1 offspring, an increased NLRP3 and IL-1β was observed in male and female offspring, however only NLRP3 in the SE male (*P* < 0.01 vs SHAM, Fig. 3a) and IL-1β in SE female (*P* < 0.05 vs SHAM, Fig. 3d) were significantly higher than the SHAM offspring. Maternal L-Carnitine treatment normalised both markers (*P* < 0.05 vs SE, Fig. 3a).

At 13 weeks, maternal cigarette SE significantly increased NLRP3 expression in female offspring (*P* < 0.01 vs SHAM, Fig. 3f); maternal L-Carnitine supplementation did not have any effect (*P* < 0.01 vs SE, Fig. 3f). Maternal smoke exposure significantly increased IL-1β level (*P* < 0.01 vs SHAM, Fig. 3g) in the male offspring, which was further increased after maternal L-Carnitine treatment (*P* < 0.01 vs SE, Fig. 3g).

### Effect of maternal SE on lung mitophagy markers in the offspring

**Fig. 4:**
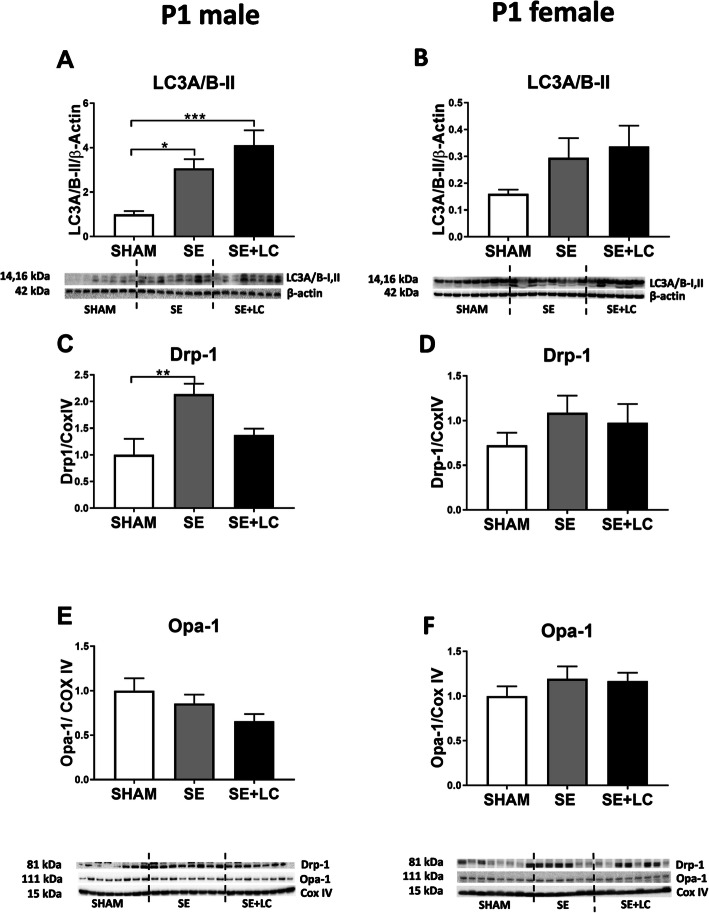
Lung LC3A/B-II, Drp-1 and Opa-1 in the offspring at P1. Protein levels of LC3A/B-II **a**, **b**, Drp-1 **c**, **d** and Opa-1 **e**, **f** in the lung of male and female offspring at P1. Results are expressed as means ± SE, (male *n =* 8, female n = 6–8). Data were analysed by one-way ANOVA followed by Tukey’s post hoc tests. * *P* < 0.05, **P < 0.01, ***P < 0.001. LC3A/B, light chain 3 microtubule-associated protein; Drp-1, dynamin-related protein; Opa-1, optic atrophy-1; SE, maternal smoke exposure; SE + LC, maternal smoke exposure with L-Carnitine supplement

At P1, total cell autophagy marker LC3A/B-II and mitochondrial fission marker Drp-1 protein levels were significantly increased in the male SE offspring (*P* < 0.05, Fig. 4a, c). Maternal L-carnitine supplementation further increased LC3A/B-II level, but normalised Drp-1 levels in the SE + LC offspring (*P* < 0.001 vs SHAM, Fig. 4a, c). No changes in autophagy and mitophagy markers were found in P1 female offspring among the 3 groups (Fig. 4b, d, e).

**Fig. 5:**
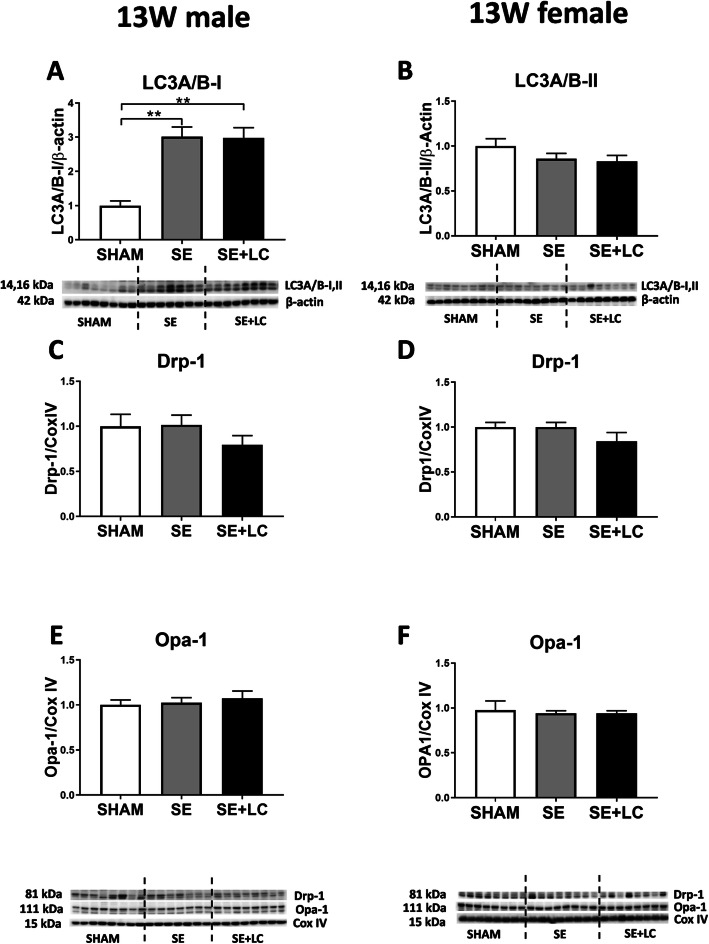
Lung LC3A/B-II, Drp-1 and Opa-1 in the offspring at 13 weeks. Protein levels of LC3A/B-II **a**, **b**, Drp-1 **c**, **d** and Opa-1 **e**, **f** in the lung of male and female offspring at 13 weeks. Results are expressed as means ±SE, (male *n =* 7–8, female *n* = 8). Data were analysed by one-way ANOVA followed by Tukey’s post hoc tests. **P < 0.01. Drp-1. LC3A/B, light chain 3 microtubule-associated protein; dynamin-related protein; Opa-1, optic atrophy-1; SE, maternal smoke exposure; SE + LC, maternal smoke exposure with L-Carnitine supplement

At 13 weeks, LC3A/B-II protein was significantly increased by maternal SE in the male offspring (*P* < 0.01, Fig. 5a) which was not affected by maternal L-Carnitine supplementation. In the female offspring, no difference in autophagy and mitophagy markers was observed among the 3 groups (Fig. 5b, d, e).

## Discussion

Maternal smoking during pregnancy is well-documented to cause long-term adverse effects on the health outcomes in multiple organs in the offspring, including respiratory, neurological, and renal systems [37]. However, the sex difference in respiratory disorders has not been broadly studied, perhaps due to the preference of using one gender to model asthma or COPD.

In this study, male offspring from the SE dams had smaller body weight from birth to adulthood, consistent with previous animal studies and humans suggesting the reproducibility and human relevance of our model [38, 39]. Maternal SE activated inflammatory NF-κB and MAPK pathways, which were more prominent in the male offspring at P1. It is well known that cigarette smoking can induce inflammation via the MAPK signalling cascade [40], reflected by increased phosphorylation of ERK and P38 [41, 42]. MAPK pathway activation can also lead to increased activation of certain transcription factors, such as NF-κB [43]. In the current study, these effects in P1 male SE offspring are likely due to the chemicals in cigarette smoke including free radicals reaching the foetus via the placenta. NLRP3 inflammasome activation in the male offspring at P1 is in accordance with other inflammatory pathways, especially NF-κB. However, only NF-κB hyperactivation was maintained at adulthood. This may be due to a lack of a second insult after birth. As NF-κB regulates acute responses to external stimuli, its innate hyperactivation may enhance the response to postnatal environmental factors, such as allergens to increase the risk of asthma or tobacco smoking to increase the risk of COPD [44]. This requires further investigation with additional modelling in the offspring.

It is not surprising to observe that female offspring are less affected by the adverse effect of maternal SE compared with the male littermates. Such a lack of response in the females is consistent with our previous observations in the brain and kidney [14, 45, 46]. In adults**,** there are known differences in innate and adaptive immune responses between males and females which might be related to immune related genes encoded upon the X chromosome being differentially expressed in males and females. Furthermore the sex hormone oestrogen can protect females from developing several diseases [46, 47].

A recent study found that inflammasomes can be the bridge between inflammation and mitochondrial function [47]. There is increasing recognition that mitochondrial dysfunction plays a key role in the development of various diseases including COPD and asthma [27, 48, 49]. Maternal smoking can induce a high level of oxidative stress in the developing foetus [50] which is persistent until adulthood to directly damage the mitochondria [14, 51]. Injured mitochondria can also induce more oxidative stress and inflammation. As such, mitophagy and autophagy are key to recycle mitochondrial fragments and eliminate defective mitochondria to maintain cellular homeostasis [52]. Increased fission maker Drp-1 and autophagosome marker LC3A/B-II in the male SE offspring at birth suggests an increased number of damaged mitochondria due to maternal SE. The fusion marker Opa-1 was not increased suggesting less mitochondrial biogenesis. At adulthood, only LC3A/B-II remained elevated, suggesting higher demand to eliminate injured cellular elements resulting from maternal SE. This may drive the development of lung disorders in the SE offspring [53]. Interestingly, mitophagy markers in the lung were not changed in the female offspring at any age, suggesting a gender-specific response to maternal SE. These results are consistent with our previous research in other organs [13].

In vivo and in vitro studies have demonstrated that L-Carnitine can prevent oxidative stress-induced injury [54–56]. In this study, maternal L-Carnitine supplementation increased birth weight in both male and female SE offspring. This suggests that L-Carnitine can ameliorate in-utero underdevelopment caused by maternal SE. Additionally, maternal L-Carnitine supplementation exhibited some anti-inflammatory effects in newborns from the SE dams, by partially suppressing NF-κB activation and NLRP3 inflammasome formation in the males, as well as MAPK pathway and IL-1β in the females. This may be due to its ability to inhibit oxidative stress induced by maternal SE in utero. However, the protection of maternal L-Carnitine supplementation during gestation at birth did not persist until adulthood, especially in the male offspring. The result of increased IL-1β in the male offspring lung, especially in the group with maternal L-Carnitine supplementation is intriguing as there was no increase in inflammasome activation. IL-1β is encoded by its own gene and produced as an in-active protein which is converted into an active protein by the inflammasome. Without a concomitant increase in inflammasome levels our results suggest at 13 weeks that either there is an increase in inactive IL-1β, not active IL-1β or the IL-1β measured by western blotting is produced external to the lungs.

The protective effects of maternal L-Carnitine supplementation have been observed in the other organs, including the brain, kidney, and liver [13, 30, 35, 57], however not in the lung as shown in this study. This is surprising, but may be explained by the limitation of how we assessed the lung in this study. Firstly, we did not collect bronchoalveolar lavage (BAL) fluid, which can provide information on inflammatory cytokine changes and inflammatory cell counts which are more direct ways to assess lung inflammation. We only used one dose of L-carnitine during pregnancy and lactation, which cannot provide the correlation between L-carnitine dose and inflammation. The multiple-dose regime needs to be tested in our future study. The hyperactivation of inflammatory signalling cascades may represent an increased ability to respond to external stimuli such as allergens or cigarette smoke, but itself may not necessarily cause lung diseases. We did not measure reactive oxygen species levels, and as such whilst it is likely that L-Carnitine supplementation acts via scavenging reactive oxygen species, we cannot definitively say this was the case.

## Conclusions

In conclusion, there are gender differences in the susceptibility to lung disorders in response to maternal smoking, with male offspring more susceptible to increased inflammatory changes. Maternal L-Carnitine supplementation during pregnancy may partially alleviate the adverse effects of maternal SE on lung health outcomes however only marked in the newborn offspring.

## Materials and methods

### Animals

The animal experiments were approved by the Animal Care and Ethics Committee at the University of Technology Sydney (ACEC#2011-313A). All protocols were performed according to the Australian National Health and Medical Research Council Guide for the Care and Use of Laboratory Animals. Female BALB/c mice (8 weeks, Animal Resources Centre, Perth, WA, Australia) were housed at 20 ± 2 °C and maintained on a 12 h light, 12 h dark cycle (lights on at 06:00 h) with ad libitum access to standard rodent chow and water. Female BALB/c mice were divided into 3 groups. The SHAM group (*n* = 11) was exposed to air in a 15 L Perspex chamber for 6 weeks prior to mating, during gestation and lactation, SE group (*n* = 21) was exposed to cigarette smoke generated from 2 cigarettes (Winfield Red, 1.2 mg nicotine; VIC, Australia) per session (5-min interval between), twice daily during the same period of time as we have previously described [46]. A sub-group of the SE dams (*n* = 12) was provided with L-Carnitine (Sapphire Bioscience Pty. Ltd., NSW, Australia) directly dissolved in drinking water (1.5 mM, SE + LC) during gestation and lactation as we have previously described [13]. This was available *ad libatum*. L-Carnitine dose was determined according to a previous publication [58]. Pregnancy was confirmed by significant weight gain 10 days after mateing. P1 mice were sacrificed by decapitation, while adult animals were sacrificed after anaesthetic overdose (Pentothal®, 0.1 mg/g, i.p., Abbott Australasia Pty. Ltd., NSW, Australia) between 9:00–12:00 h. The lungs from the offspring were collected at birth (P1) and adulthood (13 weeks) and stored at − 80 °C for later analysis.

### Western blotting

The protein levels of the markers of interest were measured in the lung, including inflammatory markers, phosphate(p)-ERK1,2 (1:2000; Cell Signalling Technology), p-JNK1,2 (1:2000; Cell Signalling Technology), p-p38 MAPK (1: 2000; Cell Signalling Technology), p- NF-κB (1:2000; Cell Signalling Technology), and autophagy markers light chain 3 microtubule-associated protein A/B (LC3A/B)-II (1:2000; Cell Signalling Technology), mitophagy fission marker dynamin-related protein (Drp)-1 (1:2000; Cell Signalling Technology) and mitophagy fusion marker optic atrophy (OPA)-1 (1:2000; Cell Signalling Technology), inflammasome marker NLRP3 (1:2000; Abcam), IL-1β (1:2000; Cell Signalling Technology), and housekeeping protein β-actin (1:10000; Cell Signalling Technology).

The lung was homogenised using cell lysis buffers for total protein and mitochondrial protein extraction through differential centrifugation as previously described [35]. Protein concentrations were measured using DC Protein assay (Bio-Rad, Hercules, CA); 15μg of proteins were separated on Ctiterion™TGX Stain Free Precast Gel (BIO-RAD, USA) and then transferred to PVDF membranes (BIO-RAD, USA), which was then blocked with TBST. The membranes were incubated with the primary antibodies, followed by horseradish peroxidase-conjugated secondary antibody (Santa Cruz Biotechnology). Protein expression was detected by SuperSignal West Pico Chemiluminescent substrate (Thermo, MA, USA) by exposing the membrane in ChemiDoc (BIO-RAD, USA). The density of the protein band was determined using Image J (National Institute of Health, Bethesda, Maryland, USA).

### Statistical methods

The results are expressed as mean ± SEM. Normality was tested by Shapiro-Wilk test prior to the statistical analysis. If the data were not normally distributed, they were log-transformed to research normality. The differences between groups were analysed by one-way ANOVA followed by Tukey’s post hoc tests (GraphPad Prism 8, GraphPad Software, CA, USA). *P* < 0.05 was considered statistically significant.

## Data Availability

The datasets herein used and analyzed are available from the corresponding author on reasonable request.

## Abbreviations

SE: Smoke exposure
COPD: Chronic obstructive pulmonary disease
ERK: Extracellular signal-regulated kinase
JNK: Jun N-terminal kinase
NLRP3: Nucleotide-binding domain and leucine-rich repeat-containing family pyrin domain containing 3
p38: p38 Mitogen-activated protein kinase
NF-κB: Nuclear factor-κB;
SE + LC: Maternal smoke exposure with L-Carnitine supplement
